# How Time, Living Situation, and Stress Related to Technology Influence User Acceptance and Usability of a Socialization Service for Older Adults and Their Formal and Informal Caregivers: Six-Month Pilot Study

**DOI:** 10.2196/54736

**Published:** 2024-10-09

**Authors:** Jasmine Pani, Letizia Lorusso, Lara Toccafondi, Grazia D'Onofrio, Filomena Ciccone, Sergio Russo, Francesco Giuliani, Daniele Sancarlo, Novella Calamida, Gianna Vignani, Tarmo Pihl, Erika Rovini, Filippo Cavallo, Laura Fiorini

**Affiliations:** 1 Department of Industrial Engineering, University of Florence, Via Santa Marta 3, Florence, 50139, Italy, 39 0552758663; 2 School of Medical Statistics and Biometry, Interdisciplinary Department of Medicine, University of Bari Aldo Moro, Bari, Italy; 3 Umana Persone Development & Research Social Enterprise, Grosseto, Italy; 4 Clinical Psychology Service, Health Department, Foundation Istituto di Ricovero e Cura a Carattere Scientifico Casa Sollievo della Sofferenza, San Giovanni Rotondo, Italy; 5 Innovation and Research Unit, Foundation Istituto di Ricovero e Cura a Carattere Scientifico Casa Sollievo della Sofferenza, San Giovanni Rotondo, Italy; 6 Geriatrics Unit, Foundation Istituto di Ricovero e Cura a Carattere Scientifico Casa Sollievo della Sofferenza, San Giovanni Rotondo, Italy; 7 Sentab, Tallin, Estonia

**Keywords:** active aging, longitudinal study, technostress, technology usability and acceptance, scaling up

## Abstract

**Background:**

Considering the growing population of older adults, addressing the influence of loneliness among this demographic group has become imperative, especially due to the link between social isolation and deterioration of mental and physical well-being. Technology has the potential to be used to create innovative solutions to increase socialization and potentially promote healthy aging.

**Objective:**

This 6-month study examined the usability and acceptability of a technology-based socialization service and explored how stress and living situation affect older adults’ and their ecosystem’s perceptions of technology, investigating cross-sectional and longitudinal differences among and across user groups.

**Methods:**

Participants were recruited in Tuscany and Apulia (Italy) through a network of social cooperatives and a research hospital, respectively. A total of 20 older adults were provided with the same technology installed on a tablet and on a smart television. The technology has three functionalities: video calling, playing games, and sharing news. Additionally, 20 informal caregivers (IC) and 13 formal caregivers (FC) connected to the older adults were included in the study. After both initial training in the use of the system (T0) and 6 months of using the system (T6), questionnaires on usability, acceptability, and technostress were filled in by older adults, IC, and FC. Nonparametric or parametric tests were conducted to investigate group differences at both time points and changes over time. Additional analyses on older adults were done to assess whether differences in usability and acceptability were related to living situation (ie, alone or with someone). Furthermore, correlation analyses were performed between usability, acceptability, and stress toward technology at T0 and T6.

**Results:**

At both T0 and T6, older adults had lower usability scores than IC and FC and higher anxiety than IC. Over time, there was a significant decrease in older adults’ attitudes toward technology score, depicting a negative attitude over time (T0 median 4.2, IQR 0.5; T6 median 3.7, IQR 0.8; Cohen *d*=0.7), while there was no change for IC and FC. At T0, those living alone had lower acceptability than those living with someone but this difference disappeared at T6. People or participants living with someone had a decline in anxiety, attitudes toward technology, enjoyment, and perceived usefulness. Stress toward technology affected usability and acceptability in the older adult group entering the study (ρ=−.85) but this was not observed after 6 months. In the IC group, stress affected trust at T0 (ρ=−.23) but not at T6.

**Conclusions:**

At the start of the study, older adults judged the system to be less usable and more stressful than did the caregivers. Indeed, at first, technostress was correlated with usability and acceptability; however, with repeated use, technostress did not influence the perception of technology. Overall, getting accustomed to technology decreased anxiety and stress toward technology.

## Introduction

Technological advances in health care research have extended the longevity of the population. However, solely increasing lifespan does not assure healthy aging or prevent age-related diseases. Older adults commonly report feeling lonely and socially isolated, feelings which are linked to decreased mental and physical health [1,2]. In Italy, 31.5% of people aged 65 years and older have limited autonomy due to health problems [3]. In 2019, it was estimated that 11.3% of older adults had depression, with the presence of comorbidity (ie, of other chronic conditions). Currently, about 65% of older adults with limited autonomy are helped by relatives, paid services, or others [3].

In this context, technological devices may play a pivotal role in supporting activities of daily living and promoting independent living. Technological devices are products that can be used to assist by increasing, maintaining, or improving functional capabilities of people with disabilities or difficulties [4]. These can range from communication devices to wheelchairs to visual or hearing aids. In recent years, there have been several research projects that have tried to develop technological devices to promote independent living and active aging [5,6]. Additionally, older adults are more likely to be experiencing or using new internet-driven personal devices such as smartphones and computers in their daily lives than before, boosted by the effects of the COVID-19 pandemic [7]. In that regard, formal caregivers (FC) and informal caregivers (IC) can play a crucial role in fostering the adoption of these technologies by older adults [8].

Contrary to stereotypes, many older adults show positive attitudes and expectations toward technological devices [9,10]. For instance, several studies have highlighted an association between social interaction through internet use and the quality of life of older adults [11]. Internet communication provides an inexpensive tool to stay connected with friends, family, and society. However, currently, the older population for the most part is not digitally experienced and decreased cognitive and physical abilities could be barriers to learning how to use technology [12,13]. Furthermore, personal susceptibility to stress could influence performance by increasing cognitive load [14].

The settings and environments in which technological devices, products, and services may be used are numerous and extend from older adults’ homes to nursing homes and hospitals. In an independent living situation, technology can be used to monitor older adults by constantly monitoring their living environment, physical activity/exercise, medicine uptake, blood pressure, and heart rate, and it can also support caregivers in their daily tasks [15-17]. In a survey conducted during the COVID-19 pandemic, more than half of older adults reported that they used technology to connect with others and that they adopted new technologies since the start of the pandemic. The study also indicated that one of the main factors that supported older adults’ willingness to learn was keeping in touch with family members, especially grandchildren [18]. The same study also highlighted that older adults living in rural areas experienced greater technological barriers to technology use. Moreover, since social isolation and loneliness are associated with higher mortality risk [19], it is important to investigate ways to increase socialization and promote social connectedness; technology could help in this matter. Indeed, although living alone has been suggested to be a risk factor for poor health, studies showed that it was a lack of social connectedness (measured by social network size), rather than the condition of living alone, that was associated with adverse health outcomes [20-22]. Furthermore, results from a systematic review and meta-analysis showed that technological interventions to support older adults in long-term care have one of the largest effect sizes in reducing social isolation and loneliness [23]. Regarding technology-supported interventions to improve well-being and socialization, from a recent systematic review [24], it was evident that the available studies had a short time span and less than half employed tailored solutions. The short time span between baseline and follow-up found in previous studies does not allow for an in-depth investigation of the effects of the use of different technological devices among older adults. Furthermore, as noted by an embedded case study [25] and reviews [26,27], adoption and acceptance are influenced by social influence from family, friends, and caregivers. It is therefore important to explore the whole ecosystem that revolves around older adults to understand the reasons behind low usage of a device or user acceptance. Additionally, soliciting a multistakeholder perspective by engaging both older adults and their caregivers in a study would be advantageous and provide further support for research outcomes.

In this context, this paper presents a service designed to increase socialization in older adults through a technological device. The study is part of the Pharaon project [28], which is a large-scale pilot under the “Digitising European Industry” strategy. The project aims to promote active aging using already available and mature platforms and technologies. The Pharaon project uses the action research method, which entails 4 cyclical actions: reflect, plan, act, observe, and then reflect again to continue through the cycle. Specifically, after deployment and data collection, reflection meetings were organized to assess how the deployment was proceeding and to better plan next actions.

For this particular socialization service, the same technology was installed on two different user interfaces (UIs): a smart television or a tablet. This technology allowed video calling between older adults (n=20) and their IC (n=20) and FC (n=13), and it offered the ability to share news/pictures and play games. The service was implemented for 6 months in the home of the older adult. The 3 cohorts were compared with each other and over time in terms of usability and acceptability in a cross-sectional and longitudinal design. Furthermore, we explored whether stress or living alone/with someone affected user perception of the older adult. Specifically, we aimed to answer the following research questions (RQ):

- RQ1: Are there any differences in acceptability and usability intergroup and between cohorts (ie, older adults, IC, FC) over time? For the older adults, are differences related to different devices?
- RQ2: Can living situation represent a discriminant factor for acceptance and usability of technology by older adults?
- RQ3: Will stress related to technology usage affect usability and acceptability as reported by older adults?

## Methods

### System Service Description

The types of technologies were selected based on feedback received from older adults and their caregivers during the needs analysis phase of the project. In the Italian pilot, the needs analysis led to the identification and deployment of two services: monitoring and socialization [29]. In this paper, we focus only on the socialization service.

The socialization service is based on Sentab technology (Sentab Estonia OÜ). The system was developed around a UI working on the web, Android, iOS, and Android TV. From a technical perspective, the Sentab backend solution was based on Enterprise Java on Jetty, open source RabbitMQ, and Redis dockers. Information was stored in a MySQL database on Ubuntu servers. The content delivery network was built on Amazon CloudFront and S3.

In the project, two UIs were used: an Android application installed on tablets (Apulia) and televisions (Tuscany) and an Android app for caregivers installed on their mobile phone. This final choice was made based on the feedback received from pilot managers as an outcome of the needs assessment. It is worth underscoring that the service was the same for both technologies—only the devices changed.

**Figure 1.**
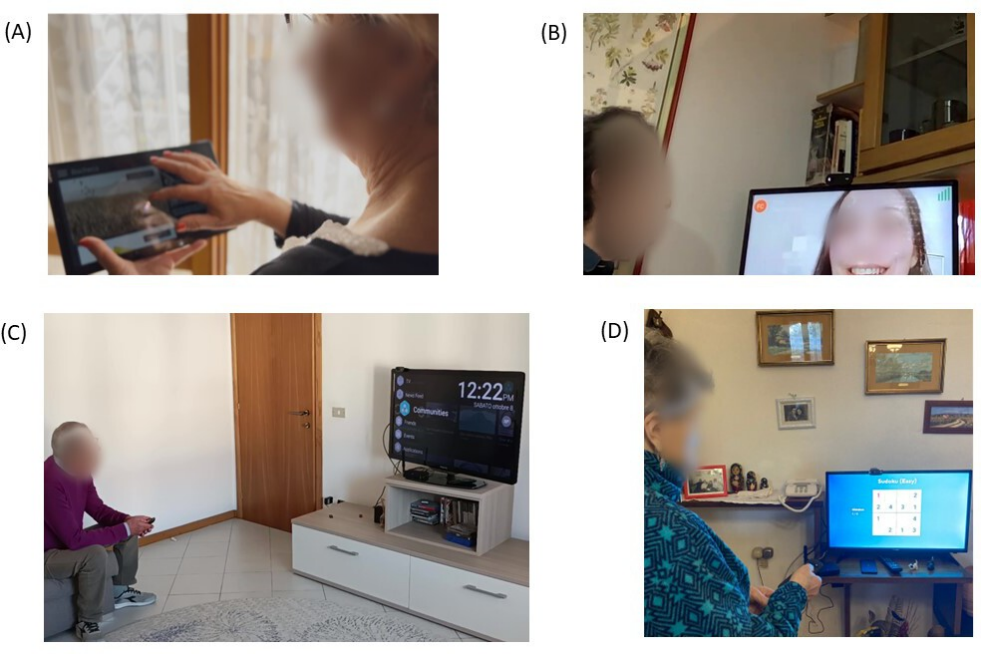
Photographs illustrating participants enrolled in the study. All participants agreed and consented to have their picture taken and used in publications. (A) Older adult checking the “stay informed” function on the tablet. (B) Older adult video calling their formal caregiver on a smart television. (C) Older adult using the smart television. (D) Older adult participant using the television game app.

The older adults could interact with the tablet using the touch screen, whereas for the television, a separate remote control was provided and Sentab was accessed using the arrows and an “OK” button (Figure 1). For both technologies (ie, tablet and television), the Sentab technology has the following functionalities: (1) a video calling function, where older adults and their caregivers could video call through the UIs; (2) a stimulating game function, where older adults can access some cognitive games (eg, sudoku, picture memory) and monitor their improvement by checking the cognitive index calculated by Sentab; and (3) a stay-informed function, where older adults and IC can access news and information shared by hospital clinicians regarding best practices for maintaining a healthy and active lifestyle.

An overview of the socialization service can be found online [30]. By connecting to their UIs, caregivers could communicate with their relative (or “assigned” older adult) and share photos or news.

### Experimental Protocol

**Figure 2.**
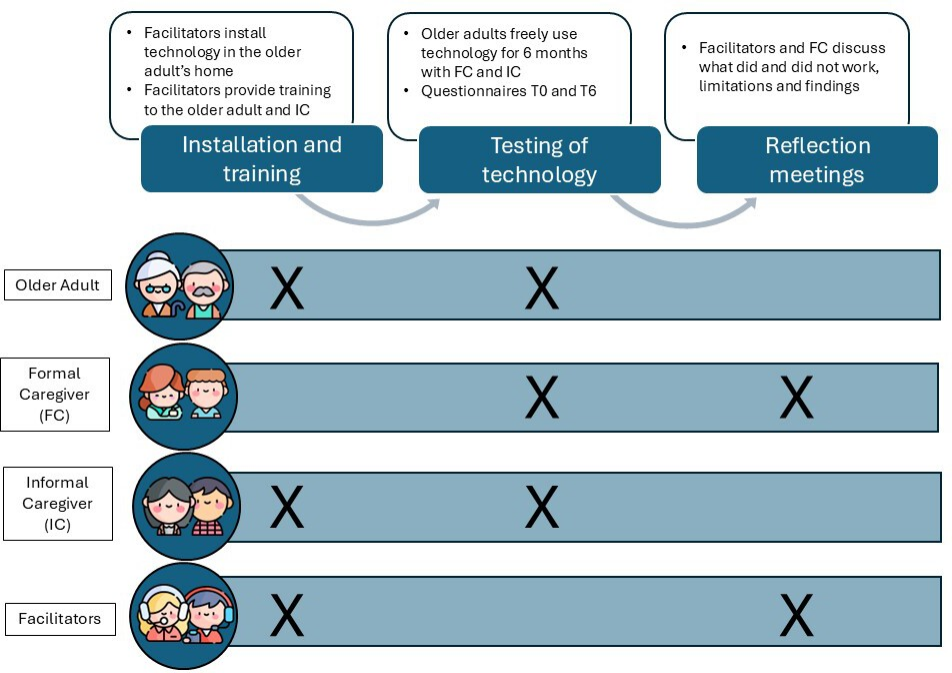
Graphical description of the experimental protocol of the 3 consecutive phases. The “X” represents when a certain cohort participated in a specific phase. Icons were downloaded from Flaticon [31]. FC: formal caregiver; IC: informal caregiver; T0: start of study; T6: end of study (after 6 months of technology use).

The experimental protocol was composed of the following phases: installation and training, testing of technology, and a reflection meeting. An overview of these phases is presented in Figure 2.

As mentioned in the System Service Description section, Tuscany and Apulia tested different devices. In Tuscany, the smart television was installed in the older adult’s home and then facilitators trained them in the use of the technology. On the other hand, in the Apulian pilot, facilitators trained the IC together with the older adult and the tablet was subsequently given to the older adult to take home and use freely. The participants could ask questions for clarification. At the end of the training, facilitators gave the participants the user manual along with their contact details.

As soon as the participants felt confident with the technology, the facilitators administered the questionnaires, providing baseline data (T0). Participants were then requested to freely use the technology and the service functionalities in their daily life. After 6 months of use (T6), they were requested to fill in the same questionnaire that was administered at T0. A description of the questionnaires is provided in the Questionnaires section.

To better understand the end users, we used an action research approach that suggests organizing reflection meetings to critically reflect on the “results of action” [32] and to underscore the lessons learned in terms of aspects that did or did not work well in the pilot. The reflection meeting was scheduled after the first 6 months of use to collect feedback and it involved meeting facilitators, facilitators, and FCs. The meeting facilitators were in charge of presenting the results, fostering discussion, and taking notes during the meeting.

The meeting was divided in three parts: presentation of the results following the first 6 months of use, discussion with the facilitators and FCs, and an online survey. After the initial presentation, the quantitative results were discussed to understand whether the facilitators and FCs expected or were surprised by these results and whether they could explain the results in a qualitative way. The discussion touched on the following: (1) the things that did and did not work properly; (2) the limitations of the technology; and (3) the findings of the study, including acceptance of the technology. After the plenary discussion, before closing the meeting, participants were asked to complete an online survey to give feedback on the main factors related to technology readiness and its acceptability; additionally, participants were asked to report what they considered to be a significant outcome from the perspective of the older adult, IC, and FC. The results of this meeting and survey were then aggregated to critically discuss the results of the questionnaires and to plan and suggest corrective actions for future tests.

### Participants

In order to answer this study’s RQs, three cohorts of participants were recruited: older adults, IC, and FC (professionals in charge of their monitoring). A total of 105 people were recruited to be part of the study: 40 older adults, 40 IC, and 25 FC. The older adults were recruited and were randomized 1:1 into either the control or intervention group. The IC and FC were divided into control and intervention groups according to their older adult’s randomization.

The inclusion criteria for the older adults were aged ≥60 years, intact cognitive status (Mini-Mental State Examination score >24), and a frailty score from well to moderately frail; exclusion criteria were presence of severe cognitive impairment and other conditions that cause memory impairment or engagement difficulties. Participants in the control group were recruited with the same inclusion and exclusion criteria; this group did not interact with the technology and was only required to fill in the sociodemographic questionnaire. In this study, we will focus on the user experience of technology use, and thus only focus on the intervention group (older adults: n=20; IC: n=20; FC: n=13).

Participants were enrolled in two Italian regions (Apulia and Tuscany) that composed the Pharaon Italian Pilot. The recruitment strategies were different across the two pilots; in Apulia, the IC was recruited first, whereas in Tuscany, the older adult was recruited first. In Apulia, the participants were recruited at the Casa Sollievo della Sofferenza research hospital (San Giovanni Rotondo, Foggia, Italy) in different clinical units and at the University of the Third Age, and no participants were cognitively or medically compromised. In Tuscany, the participants were recruited among people that were already accessing home services provided by the Umana Persone S.r.l. (Grosseto, Italy) network of social cooperatives. The research was approved by the Comitato Etico Clinico, Azienda USL Toscana Sud Est on July 22, 2021 (Prot. 2021/000227), and by the Azienda USL Toscana Centro on October 18, 2022 (Prot. 2022/22131_spe). Furthermore, the two pilot sites used the technology either on a tablet or a smart television according to guidelines and feedback acquired in the needs analysis [29].

### Ethical Considerations

The ethical committee of the Casa Sollievo della Sofferenza research hospital approved the research on June 14th, 2021, with protocol number 89/CE. All participants read and signed the informed consent form before entering the study. The collected data were anonymized and no compensation was provided to participants.

### Facilitators

Following the action research framework and to better discuss and reflect on the RQs, we also involved the facilitators, who have different professional backgrounds ranging from engineers to cooperative managers to health professionals, and whose main task was to ease the use of technology in all 3 cohorts and solve any problems that arose during the experimentation. Additionally, facilitators installed the technology and delivered the training to the intervention group and administered the questionnaires.

A total of 20 participants joined the reflection meeting: 5 participants from the University of Florence, who acted as meeting facilitators; 3 participants from the Apulian pilot site (Casa Sollievo della Sofferenza Research Hospital); and the remaining 12 participants were from the Tuscan pilot site (Umana Persone network of social cooperatives). Only 8 facilitators (3 from the Apulian pilot and 5 from the Tuscan pilot) agreed to fill out the final survey.

### Questionnaires

To gather information about the participants’ characteristics, sex, age, education, and living situation/environment, a semistructured interview was conducted at the beginning of the study. The Mini-Mental State Examination [33] was administered to assess the older adults’ cognitive status.

Usability was measured with the system usability scale (SUS) [34] (Italian version [35]), which is composed of 10 items on a 5-point Likert scale where 1 stands for “strongly disagree” and 5 stands for “strongly agree.” Items 2, 4, 6, 8, and 10 need to be reversed. The total SUS score is obtained by adding all the score contributions and multiplying the sum by 2.5. The SUS ranges from 0 to 100; scores lower than 68 are considered below average.

Acceptance was evaluated through the Almere model questionnaire (AMQ) [36], which is composed of 41 items on a 5-point agreement Likert scale. The AMQ is composed of 12 constructs created by combining specific items: anxiety (ANX), attitude toward technology (ATT), facilitating conditions, intention to use (ITU), perceived adaptiveness, perceived enjoyment (ENJ), perceived ease of use, perceived sociability, perceived usefulness (PU), social influence, social presence, and trust (TRUST). We investigated 6 of these 12 constructs: ANX, ATT, ITU, ENJ, PU, and TRUST. Note that ANX is reversed, therefore a higher ANX score translates to lower levels of anxiety.

To measure the quantity of perceived stress related to technology use (technostress), the Perceived Stress Scale [37] (Italian version [38] adapted as in [39]) was administered. The test comprises 10 items with a 0‐4 Likert scale with 0 meaning “never” and 4 being “very often.” Items 4, 5, 7, and 8 have reverse scoring. The total score is calculated as the sum of the single item contributions. A total score from 0-13 is considered low stress, 14-26 is moderate stress, and ≥27 is high stress.

At T6, participants were also asked to estimate how frequently they used technology by asking how many times they used it per day and per month.

A schematic overview of when and to whom the questionnaires were administered is presented in Table 1.

**Table 1.** Overview of the interview topics and questionnaires including when they were conducted and with whom.

	T0^a^	T6^b^
**Semistructured interview**		
Age, sex, education, digital skills	OA^c^, IC^d^	—^e^
Living situation (alone/not alone) and living environment (urban/rural)	OA	—
Technology usage question	—	OA
**Questionnaires**		
Mini-Mental State Examination [33]	OA	—
System usability scale [34], Almere model questionnaire [36], and technostress [39]	OA, IC, FC^f^	OA, IC, FC

a T0: start of the study.

b T6: following 6 months of technology use.

c OA: older adult.

d IC: informal caregiver.

e Not applicable.

f FC: formal caregiver.

### Statistical Analysis

#### Overview

We performed statistical analyses to evaluate longitudinal intragroup and cohort differences in usability and acceptability among the older adults, IC, and FC. Moreover, we investigated the effect of the older adults’ living situation (ie, living alone or with someone) and stress related to technology on usability and acceptability. Each analysis is described in detail in the following subparagraphs. For each questionnaire, we calculated reliability with Cronbach α. Given the low sample size, a value of ≥.6 was deemed acceptable [40,41]. Effect sizes of significant results were calculated with Cohen *d*. In the statistical tests performed, a *P* value <.05 was considered statistically significant. Statistical analyses and graphical illustrations were performed using RStudio [42] (version 4.2.3; Posit team).

#### Differences in Usability and Acceptability

Data for SUS and AMQ were checked for normality using the Shapiro test. For changes over time in each cohort (intragroup), we checked for normality the distribution of the differences between T0 and T6. If the data were normally distributed, we performed a two-tailed paired *t* test to examine differences over time, otherwise a paired Wilcoxon signed-rank test was preferred. For older adults, additional analyses were performed to investigate differences between pilot sites.

A two-way mixed ANOVA was used to compare the scores for older adults, IC, and FC at T0 and T6, as well as changes over time (cohort differences). For older adults and IC, the models were also repeated including age and sex in the model to account for demographic differences in the two cohorts. If there was a statistically significant effect, post hoc two-tailed pair-wise *t* tests were Bonferroni corrected.

#### Effects of Living Situation on Usability and Acceptability in Older Adults

For older adults, a two-tailed *t* test was performed to investigate the effects of living situation (ie, alone or with someone) at T0 and T6, and a two-tailed paired *t* test was used to assess change over time between T0 and T6.

#### Effects of Technostress on Usability and Acceptability

For the older adult, IC, and FC groups, correlations were performed between technostress, SUS, and AMQ results. Data for technostress, SUS, and AMQ were checked for normality using the Shapiro test. If data were normally distributed, the correlation was Pearson, otherwise it was Kendall.

## Results

### Participant Characteristics

The demographic and cognitive characteristics as well as technostress at T0 and T6 for older adults and IC are presented in Table 2.

Participants on average used the technology 4 times per week. Overall, 85% (17/20) of the older adults lived in an urban area, and 60% (12/20) of the older adults lived with someone, of which 42% (5/12) lived with their IC. The older adults’ associated FC were predominantly women (69%, 9/13). The FC were of various professional backgrounds including psychologists, nurses, and care workers. No other demographic information was collected for FC.

**Table 2.** Demographic and cognitive characteristics and technostress in the older adult and informal caregiver groups.

Characteristics		Older adults (n=20)	Informal caregivers (n=20)
Age (years), mean (SD)		77.15 (7.07)	47.85 (13.10)
Sex (women), n (%)		14 (70)	9 (45)
**Education, n (%)**			
	Primary education	13 (65)	0 (0)
	Secondary education	5 (25)	10 (50)
	Tertiary education	2 (10)	10 (50)
Mini-Mental State Examination score, median (IQR)^a^		26.45 (6.55)	—^b^
**Technostress, median (IQR)^a^**			
	T0^c^	13 (11.50)	7.00 (9.25)
	T6^d^	10.50 (3.50)	5.50 (5.50)

a Median values and IQR are presented when the variables were not normally distributed.

b Not applicable.

c T0: start of the study.

d T6: following 6 months of technology use.

### Differences in Usability

#### Intragroup and Pilot Site Differences in Usability

The Cronbach α for the SUS in all groups was higher than .6 at both T0 and T6.

**Figure 3.**
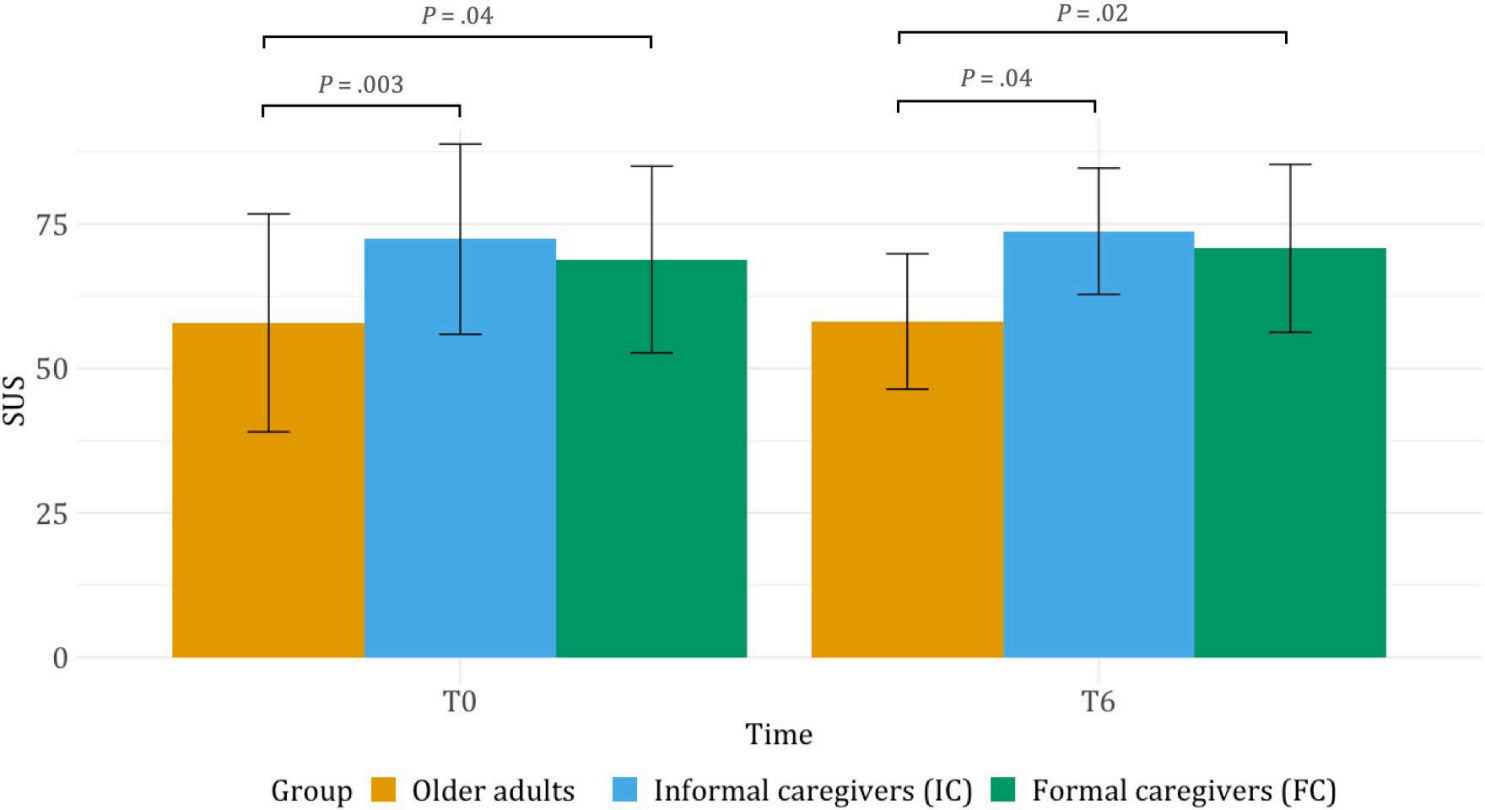
SUS score in older adults, informal caregivers, and formal caregivers at T0 and T6. The error bars represent the standard deviation for each group. The brackets highlight statistical differences between groups. Note that the difference between older adults and FC at T0 and T6 was not significant after post hoc Bonferroni correction. SUS: system usability scale.

In the older adults group, the distribution of the difference over time in the whole sample was not significantly different from a normal distribution, and normality was assumed. The mean SUS score increased slightly over time; however, this increase was not significant (Figure 3).

Both the Apulian and Tuscan pilot differences over time were distributed normally; still, there was no significant change over time. The mean of the Tuscan pilot increased and the standard deviation decreased, whereas in the Apulian pilot, mean SUS score decreased from T0 to T6 (Apulia T0: 70.8, SD 12.2 vs T6: 66.5, SD 4.7; Tuscany T0: 45.0, SD 15.3 vs T6: 49.8, SD 10.6).

In the IC group, the distribution of the differences over time in the whole sample was not significantly different from a normal distribution, whereas it was normally distributed for the FC group. The SUS score did not change over time in both groups (Figure 3).

#### Cohort Differences in Usability

A 2-way mixed ANOVA with group (older adult, IC, and FC), time, and group × time interaction showed an effect of group on SUS score, but not of time or group × time interaction. Post hoc pair-wise *t* test showed a significant difference between older adults and IC at T0 (older adults: mean 57.9, SD 18.9; IC: mean 72.4, SD 16.5; *P*=.003; Cohen *d*=0.8) and T6 (older adults: mean 58.2, SD 11.7; IC: mean 73.8, SD 10.9; *P*=.001; Cohen *d*=1.4), and between older adults and FC both at T0 (FC: mean 68.8, SD 16.2; *P*=.04; Cohen *d*=0.6) and T6 (FC: mean 70.8, SD 14.6; *P*=.02; Cohen *d*=0.9) (Figure 3). At both time points, the older adults had significantly lower SUS scores than the IC and FC. The difference between older adults and IC was not statistically significant when considering age and sex in the model (group × time interaction *P=*.86). The difference between older adults and FC was not statistically significant after post hoc Bonferroni correction (T0: *P*=.13; T6: *P*=.06).

### Differences in Acceptability

#### Intragroup and Pilot Site Differences in Acceptability

For the older adults and IC groups, Cronbach α for each construct of the AMQ was ≥.7 at T0 and T6. In the FC group, at T0, Cronbach α was greater than .6 for all constructs except PU, for which it was .2. At T6, reliability was greater than .7 for all AMQ constructs.

In the older adults group, for ITU and TRUST, the distribution of the difference over time was not different from a normal distribution. The distributions of the difference over time in the other constructs (ie, ANX, ATT, ENJ, TRUST) were not normal.

**Figure 4.**
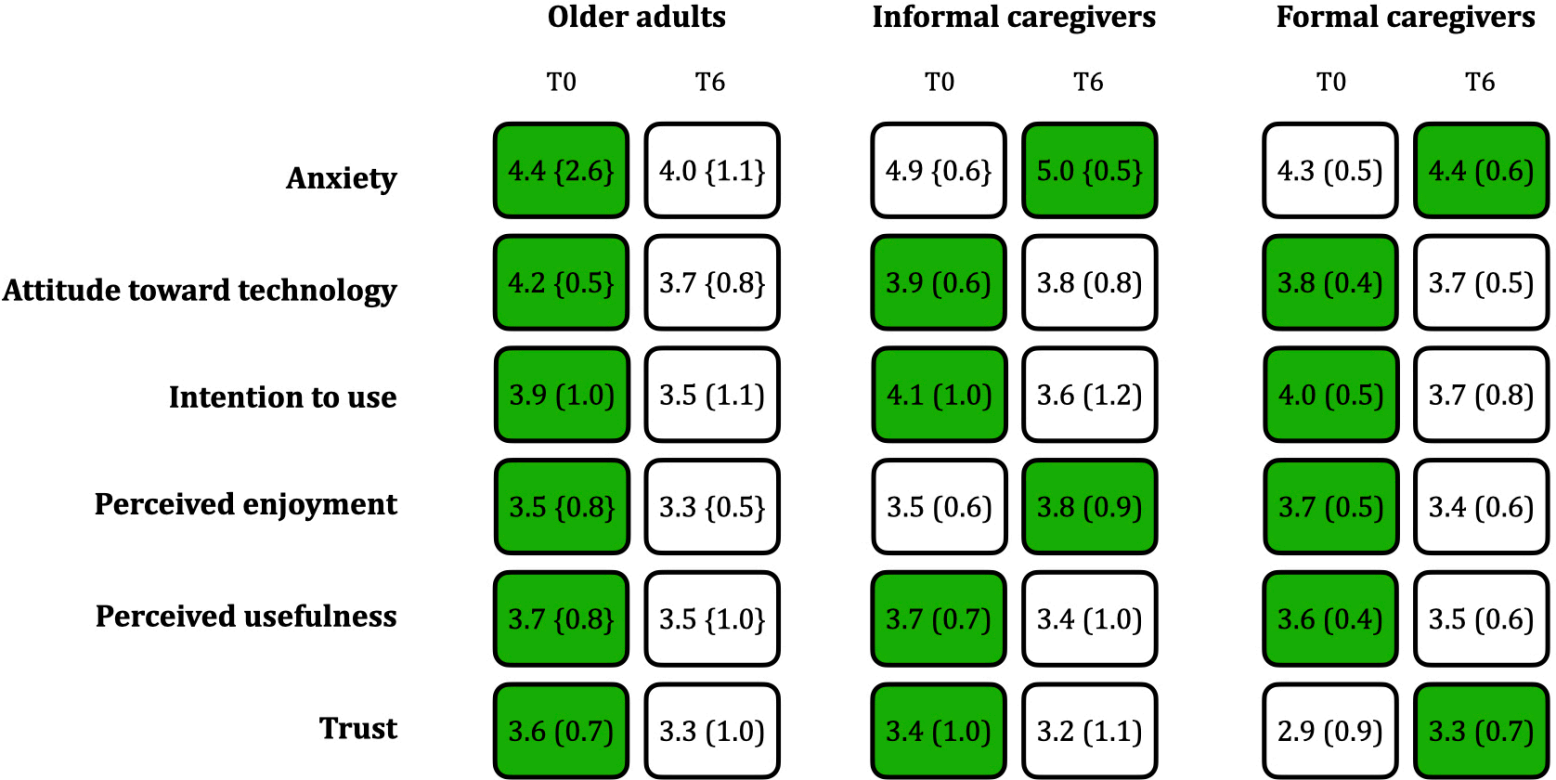
Mean values and standard deviations in parentheses for all acceptability constructs for older adults, informal caregivers, and formal caregivers at T0 (start of the study) and T6 (after 6 months of technology use). The green background highlights the higher value for each row, either at T0 or T6. Note that values are mean (SD) or median {IQR} according to the normality of the differences over time.

ATT significantly decreased (*P*=.01; Cohen *d*=0.7), whereas the other constructs did not significantly increase or decrease over time (Figure 4). In the Apulian pilot, there was a significant decrease in ANX over time (T0: 4.9 and T6: 4.3; *P*=.03; Cohen *d*=1.0) but no other significant changes in constructs were found. No changes in AMQ constructs over time were found in the Tuscan pilot (see Table S1 in Multimedia Appendix 1 for mean and median values).

In the IC group, the distribution of the differences was different from a normal distribution for ANX, but the distribution of the differences for other constructs could be assumed to be normal. In the FC group, the distribution of the differences in all constructs was not different from a normal distribution. There was no significant change over time in any construct in either the IC or FC group (Figure 4).

#### Cohort Differences in Acceptability

A 2-way mixed ANOVA with group (older adult, IC, and FC), time, and group × time interaction showed an effect of group on ANX, and time for ATT and ITU. No other group, time, or group × time interactions were found.

For ANX, post hoc pair-wise *t* test showed a significant difference between older adults and IC at T0 (*P*=.005; Cohen *d*=0.7) and T6 (*P*=.002; Cohen *d*=1.2), with older adults having higher anxiety than IC. The significant difference in ANX between older adults and IC remained after post hoc Bonferroni correction but disappeared when accounting for age and sex in the model (T0: *P*=.20; T6 *P*=.68). For ATT, there was a significant effect of time for only the older adults, as discussed in the Differences in Acceptability section (Figure 4). There was no effect of time on ITU for any group.

### Effects of Living Situation on Usability and Acceptability in Older Adults

For usability, at T0 and T6, there was no difference between those living alone and those living with someone. There was no significant change over time in SUS score in either living situation.

For acceptability, at T0, those living alone had significantly lower AMQ constructs than those living with someone (ANX *P*=.02, Cohen *d*=1.1; ATT *P*=.04, Cohen *d*=0.9; ITU *P*=.01, Cohen *d*=1.3; ENJ *P*=.03, Cohen *d*=1.0; PU *P*=.01, Cohen *d*=1.4; TRUST *P*=.01, Cohen *d*=1.3). After 6 months, the difference disappeared (see Table S2 in Multimedia Appendix 1 for mean and median values).

In older adults living alone, there was no change in AMQ constructs over time, whereas in those living with someone, ANX, ATT, ENJ, and PU declined over time (ANX *P*=.03, Cohen *d*=0.7; ATT *P*=.02, Cohen *d*=0.8; ENJ *P*=.03, Cohen *d*=0.7; PU *P*=.04, Cohen *d*=0.6; see Table S2 in Multimedia Appendix 1 for mean and median values).

### Effects of Technostress on Usability and Acceptability

#### Older Adults

**Figure 5.**
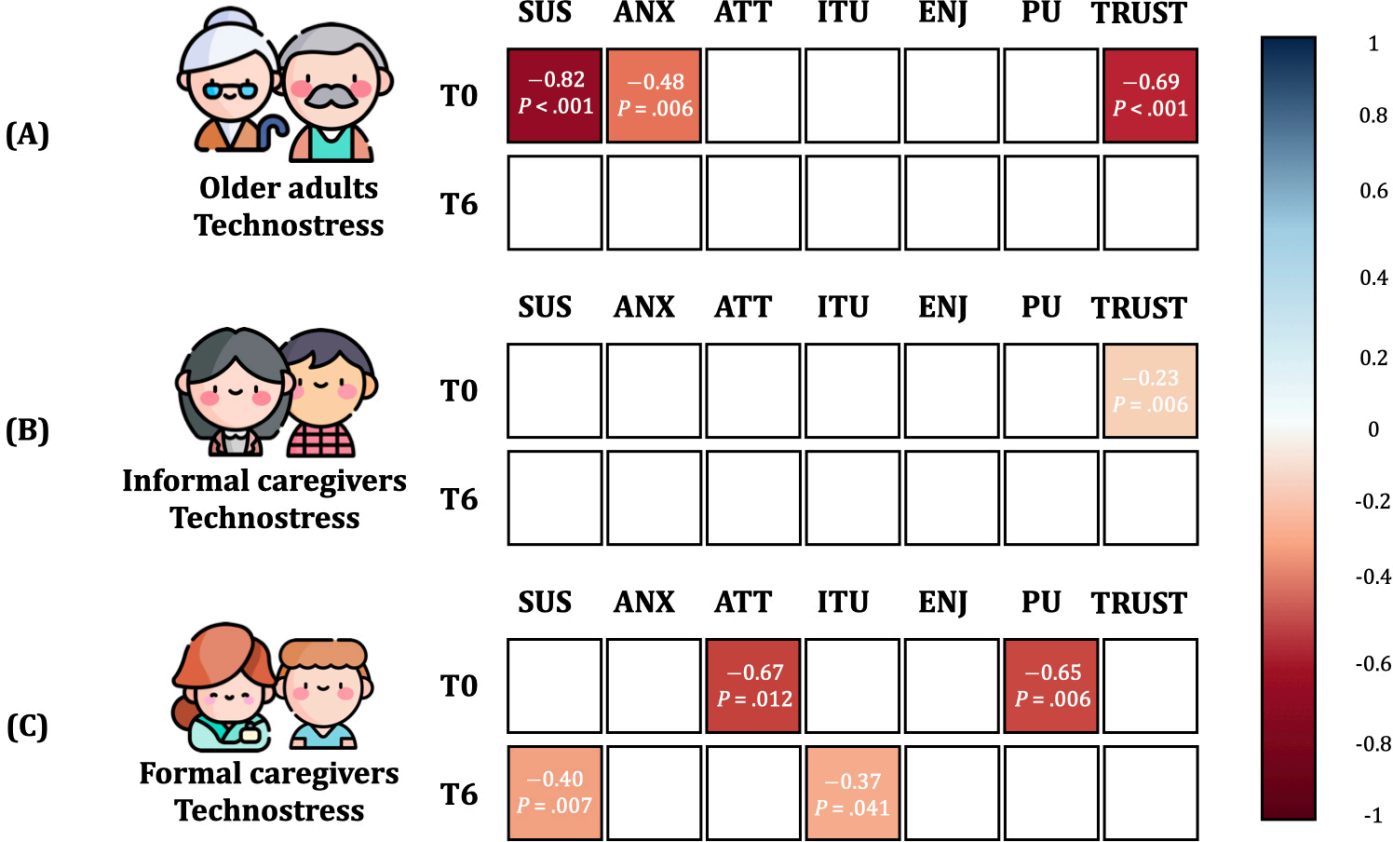
Correlations between stress toward technology (technostress) and user experience (SUS and AMQ constructs) in (A) older adults, (B) informal caregivers, and (C) formal caregivers at T0 (start of study) and at T6 (following 6 months of technology use). Only statistically significant results are presented. AMQ: Almere model questionnaire; ANX: anxiety; ATT: attitude toward technology; ENJ: enjoyment; ITU: intention to use; PU: perceived usefulness; SUS: system usability scale; TRUST; trust.

At T0, the ANX and ITU constructs of AMQ were not normally distributed, thus Kendall correlation was used; the same applied to technostress, ITU, and PU at T6. We found that technostress was highly negatively correlated with SUS score and TRUST and moderately negatively correlated with ANX (Figure 5A). After 6 months, technostress was not associated with SUS score or AMQ (Figure 5A).

#### Informal Caregivers

In the IC group, at T0, technostress, ANX, ITU, PU, and TRUST were not normally distributed; at T6, ANX, ITU, and PU were not normally distributed. At T0, technostress was significantly negatively correlated with TRUST, yet technostress was not associated with SUS score or other acceptability constructs (Figure 5B). After 6 months, the significant correlation between technostress and TRUST was no longer present (Figure 5B).

#### Formal Caregivers

ENJ and PU at T0 and technostress, SUS, ANX, and TRUST at T6 were not normally distributed. At T0, there was a significant moderate negative correlation between technostress and ATT and between technostress and PU (Figure 5C). At T6, technostress was moderately negatively correlated with SUS score and with ITU; there were no other significant correlations between technostress and AMQ constructs (Figure 5C).

### Results of the Reflection Meeting With Facilitators

#### Overview

The reflection meeting lasted 2 hours and began with the presentation of the results, followed by a discussion. The discussion focused on the personal experiences and perceptions that facilitators and FC had, then touched upon aspects that did and did not work, technical limitations, and findings. The collected feedback was transcribed and combined with the responses collected by means of a questionnaire that dealt with similar topics. These arguments were aggregated and then summarized into concepts that could limit the use of technology. The identified barriers are summarized in the following sections and were used to discuss the quantitative results.

#### Digital Skills

All facilitators highlighted that in Italy, the educational level and digital skills of older adults are low, a finding confirmed by other studies [43]. These contextual factors could represent a barrier at the start of the study. Particularly, facilitators noticed differences in the ATT between older adults aged 65-70 years old and those aged ≥70 years. They also stated that the younger cohort of older adults was more skilled and “ready” to accept the technology compared to the older group. Indeed, the younger cohort was more likely to have a smartphone and use apps for SMS text messaging (eg, WhatsApp).

#### Recruitment

During the meeting, the facilitator highlighted some difficulties they had in recruiting the older adults and IC. As mentioned in the Methods section, the two pilots used two different strategies for recruiting participants. In Apulia, facilitators first recruited the IC by asking if they would like to participate in the study with her/his older relatives. Conversely, in Tuscany, the older adults were the first to be recruited. Facilitators also noticed that older adults were more likely to agree to be part of the study when they were part of domiciliary services (Tuscany pilot) or if they already had a relationship of trust with them. During the meeting, the facilitator exchanged ideas and strategies to optimize recruitment in the pilots.

#### Technology Reliability and Ease of Use

Technology reliability affects the user experience; indeed, facilitators indicated that when there were issues with the technology, such as “internet connection issues” or “other bugs,” this had a negative influence on the experience, whereas ease of use was one of the key factors that positively influenced the overall user experience. During the meeting, the Tuscan facilitators reported some difficulties for older adults in accessing the service because the user must switch the “input” source on the television before using the Sentab technology, and the remote needed to be pointed directly at the Sentab box instead of the television. This procedure was considered confusing and complex. It was also noticed that after training, although the older adults were able to make the input switch and were able to use the technology, they tended not to use it independently. This feedback reflects the usability data; indeed, the older adults’ usability was lower in the Tuscan pilot than in the Apulian one. However, older adults who were more motivated in using the technology used it without problem; this was the case for a 90 year old enrolled in Tuscany that loved the sudoku game. On the contrary, the Apulian facilitators did not experience any barriers related to technology use (ie, tablets). Indeed, they confirmed that it was the right technology for the older adult population enrolled in the study. Specifically, older adults in Apulia appreciated the size of the digital keyboard letters they were using.

#### Training

Facilitators observed and reported that older adults had some prejudice and mistrust of new technologies; therefore, we believe that the initial contact and explanation of the project must be effective. Facilitators noticed that older adults often forgot the instructions for the technology, therefore a more effective training process should be composed of multiple training sessions. Indeed, facilitators decided to call back the older adults after the initial training to refresh the instructions during the first weeks of testing.

#### Cognitive and Living Situation Profile

Facilitators highlighted that the older adults’ cognitive profile may impact technology acceptance. Additionally, the older adults’ cognitive profile may also impact their understanding of the questionnaire; indeed, the facilitators noticed that sometimes the older adults did not fully understand the questions. Facilitators also noticed some differences in technology perceptions linked to their living situation; namely, that the older adults living alone were overall more enthusiastic and perceived the technology as more useful than those living with someone, qualitatively confirming the research hypothesis.

#### Engagement

Facilitators highlighted that engagement and use of the technology were related to finding intrinsic motivation and perceiving the added value the technology may have on the older adult’s life. Additionally, another suggestion from the facilitator was to foster interaction with other people using the same technology. As a matter of fact, older adults appreciated the presence of the operator and if and when they were unable to play a game, they called the facilitators. In Apulia, the facilitators re-explained the game to one older adult, and she really enjoyed the technology. Therefore, if the older adults are appropriately stimulated, they consequently use the technology. For instance, in the case of the “stay informed” function, the periodical publication of news acted as a “stimulus,” so the older adults were more likely to read it. Additionally, facilitators were concerned that the technology could be used by the older adults not solely for the socialization service, but more as a means to foster the relationship with the caregiver. Indeed, in projects such as Pharaon, when the participant is recruited, he/she experiences more physical presence and connection with the operator compared to the usual home assistance service. Therefore, conceivably, the older adults tend to use the technology only with the operator and not independently because they do not know how to use it without assistance or do not have a real reason to use it.

## Discussion

This paper aims to investigate the role of time spent using technologies, living situation, and technostress in older adults and their caregivers that were using a technological device aimed at increasing older adults’ socialization.

### Principal Results and Comparison With the Literature

The first research question aimed to investigate the role of time in technology perception intragroup and between cohorts of participants (RQ1). As for the acceptance evaluated intragroup, except for ATT, the differences reported in Figure 4 are just trends, as they are not statistically significant. It is worth mentioning that the older adult values for ITU (T0=3.88; T6=3.53) and ATT (T0=4.20; T6=3.65) were comparable to the intention to use and attitude measured in a related work (ITU=3.34; ATT=3.73) [44]. Moreover, values for ENJ, TRUST, and PU were similar to those of an older Chinese sample, demonstrating that the values we reported are aligned with the literature [45] even though the populations are geographically different. Note that we evaluated ITU and ATT with different models, but these constructs had comparable items. Additionally, in this study we observed a decrease of ATT for all 3 cohorts of participants over time. This result is aligned with the feedback collected during the reflection meeting; indeed, the facilitators emphasized how engagement is strongly interconnected with the personal intrinsic motivation that leads to long-term use of the technology. It was also noted that the FC or IC contacting the older adults through the technology (whether video calling or news sharing) helped in keeping the participant engaged. The higher value of ATT at T0 may be due to the older adults’ initial high expectations. Nevertheless, despite the decrease in ATT, the participants stated they used the system on average 4 times per week.

It is also worth noting that we observed a higher TRUST value for the FC after use compared to the other two cohorts. Higher trust is important because trust is strictly linked with the use of technology. These results are aligned with the feedback collected during the reflection session that highlighted some participants’ mistrust in technology use at the beginning of the study, during the training session.

Usability was rated differently between older adults and their caregivers, who on average rated the system higher through SUS. These results are aligned with a recent survey [10] that highlighted a different attitude and expectation toward technology according to age group. Indeed, the statistical difference between the older adults and IC group disappeared when including age and sex as controlling variables. Other than age and sex, there could be other factors that contributed to this result, such as the older adults’ digital competence. Furthermore, as described in the Technology Reliability and Ease of Use section, it emerged that in Tuscany, the system was perceived as more difficult to use by older adults, and they needed the caregivers’ support to use it. Nevertheless, despite the lower values, the usability for older adults increased over time, though not significantly. This suggests that older adults overcame their initial technical barrier and learned how to use the system, and this was independent of their living situation. Facilitators also noted that technical problems that occurred during the trial or problems surrounding the older adults’ experience of using the technology negatively impacted technology acceptance, as also observed by Peek et al [27].

In the Apulian pilot, there was a decrease in ANX, which translated into an unexpectedly higher anxiety perception (see Table S1 in Multimedia Appendix 1 for mean and median values). On the other hand, in the Tuscan pilot, there was no significant decrease or increase in acceptability. However, it is also worth noting that the recruitment of the older adults in the Tuscan cohort was performed among those using domiciliary care services and thus included older adults with higher frailty and lower Mini-Mental State Examination score compared to the older adults in the Apulian pilot site, which might influence perception of the technology. This was also brought up in the reflection meeting. The difference in user experience between the 2 pilots could be related to the different types of technology (television and tablet) selected at the beginning of the study or to the slightly different profile of the 2 cohorts of older adults.

RQ2 aimed to investigate the role of living situation on acceptance; we found that those living alone had significantly lower AMQ constructs compared to those living with someone. However, this difference disappeared at T6. It should also be pointed out that those living with someone had decreased ANX, ATT, and PU over time, indicating that the anxiety toward the system increased, and the usefulness and attitude toward technology lowered, suggesting that having someone helping them use the system may remove the perceived usefulness of the technology. This finding could suggest that living situation may be a barrier at the beginning of the study: people that are living alone may be more skeptical at the beginning, whereas people who are living with someone may have had higher expectations, yet the reality after 6 months of use was disappointing, which lowered the acceptability of the system.

Finally, this paper aimed to investigate how stress related to technology may affect the acceptance and usability of a certain technology (RQ3). The results highlighted a strong correlation between technostress and usability, anxiety, and trust in the older adults at T0 (Figure 5A) but all the correlations disappeared at T6. The results obtained at T0 are aligned with our previous findings [39], where we highlighted a link between perceived stress and related acceptance. However, these results may suggest that technostress could be a barrier only at the beginning of the study; as soon as the older adult becomes familiar with the technology, the link between perceived stress and related acceptance disappears. To mitigate the effects of stress at the beginning of the study, proper training sessions should be organized. These sessions ought to be devoted to successfully teaching participants how to use the technology and recall the functionality of the system after the training session, adapting the training to the participant’s cognitive and educational level. As remarked by facilitators, oftentimes older adults forgot how to use the devices and tended not to use them unless facilitators retaught them how to use the technologies. Indeed, well-conceived training is a key feature of success and critical for technology acceptance.

At T0, for the IC group, higher perceived stress was a barrier to trusting technology, and in the FC group, stress affected attitude and usefulness of technology, possibly because of fear of substitution or the worry that the technology may involve extra work for them [46-48]. Nevertheless, the correlations were not significant at T6 (Figure 5B,C). This highlights how technology-related stress for the proposed socialization service can be a barrier to technology acceptance; as caregivers can influence older adults’ perception of technology, it is crucial to include them in the study to get a multistakeholder perspective to support and reassure their older adults and promote a positive attitude toward technology.

### Limitations

The main limitations of this work are the sample sizes and the duration of the test phase (6 months), as well as the different recruitment strategies carried out at the 2 different sites. As for the sample size, we aim to increase the number of participants in the 2 pilots while tracking their cognitive abilities, thus evaluating the effect of this variable on the use of technology. Further studies should also be planned to extend the duration of the testing phase, given that our preliminary results at 6 months seemed to indicate that it was not long enough to get information regarding the impact of this service in real life. This is also of interest to policy makers who are coping with staff shortages and increasing health care expenses. Another encountered limitation was the different recruitment process between pilot sites and the lack of randomization for the technologies. These factors can all contribute to different results between samples; researchers should aim to standardize procedures as much as possible to obtain generalizable results. Future studies should also investigate the factors that may influence the low use of the technology and come up with countermeasures to encourage the use of technology, which can also have an impact on acceptability [27].

### Conclusions

This paper investigates the role of time, living situation, and stress related to technology use on the usability and the acceptance of a socialization service. This paper presents the results collected after 6 months of use considering a multistakeholder perspective. In this study, we found that the older adults had higher stress and anxiety toward technology than the caregivers. Nevertheless, getting accustomed to technology over 6 months of use removed this initial barrier. It is also important to consider the living situation of the older adults as those living alone had lower acceptability than those living with someone, which could suggest an increased resistance to change. However, counterintuitively, the older adults living with someone had a decrease in enjoyment, usefulness, and attitudes toward technology, possibly because living with someone limits the need of the older adults to socialize with others. The reflection meeting with the facilitators qualitatively highlighted demographic barriers in the use of technology that should be further evaluated quantitatively.

## Supplementary material

10.2196/54736 Multimedia Appendix 1 Supplementary materials.

## Abbreviations

AMQ: Almere model questionnaire
ANX: anxiety
ATT: attitude toward technology
ENJ: enjoyment
FC: formal caregiver
IC: informal caregiver
ITU: intention to use
PU: perceived usefulness
RQ: research question
SUS: system usability scale
T0: time point 0 (beginning of the study)
T6: time point 6 (6 months from baseline)
UI: user interface
