# Supplementary material for: How Time, Living Situation, and Stress Related to Technology Influence User Acceptance and Usability of a Socialization Service for Older Adults and Their Formal and Informal Caregivers: Six-Month Pilot Study
Source: JMIR Aging. 2024 Oct 9;7:e54736. doi: 10.2196/54736 (PMC11560862; doi:10.2196/54736)
Supplement: Multimedia Appendix 1 [file aging-v7-e54736-s001.pdf]

Original Paper – Supplementary material

How time, living conditions and stress related to technology influence user acceptance and usability of a socialization service for older adults and their formal and informal caregivers: a six-month pilot study

Jasmine Pani [1], Letizia Lorusso [2], Lara Toccafondi [3], Grazia D'Onofrio [4], Filomena Ciccone [4], Sergio Russo [5], Francesco Giuliani [5], Daniele Sancarlo [6], Novella Calamida [3], Gianna Vignani [3], Tarmo Phil [7], Erika Rovini [1]*, Filippo Cavallo [1], Laura Fiorini [1].

[1]Department of industrial engineering, University of Florence, Florence, Italy
[2] School of Medical Statistics and Biometry, Interdisciplinary Department of Mericine, University of Bari Aldo Moro, Bari, Italy
[3] Umana Persone Development & Research Social Enterprise, Grosseto, Italy
[4] Clinical Psychology Service, Health Department, Foundation IRCCS Casa Sollievo della Sofferenza
[5] Innovation and Research Unit, Foundation IRCCS Casa Sollievo della Sofferenza, San Giovanni Rotondo, Foggia, Italy
[6] Geriatrics Unit, Foundation IRCCS Casa Sollievo della Sofferenza
[7]Sentab, Tallin, Estonia

*Corresponding author
erika.rovini@unifi.it

Supplementary Table 1. Mean and median Almere model questionnaire (AMQ) construct scores at T0 and T6 in older adults, divided by Pilot

	Apulia				Tuscany			
	T0 (N=10)		T6 (N=10)		T0 (N=10)		T6 (N=10)	
	Mean/median	SD/IQR	Mean/median	SD/IQR	Mean/median	SD/IQR	Mean/median	SD/IQR
ANX	4.9/5.0	0.2/0.0	4.3/4.1	0.6/1.2	2.8/2.4	0.9/1.3	3.4/3.5	1.0/1.2
ATT	4.5/4.3	0.4/0.6	3.8/4,2	0.7/1.0	3.9/3.8	0.5/0.3	3.5/3.7	0.5/0.6
ITU	4.5/5.0	0.7/0.9	4.2/4.7	1.0/1.3	3.3/3.3	1.0/1.4	2.8/3.0	0.6/0.3
ENJ	3.8/3.6	0.8/1.1	3.3/3.2	0.7/0.9	3.4/3.3	0.7/0.9	3.2/3.3	0.4/0.5
PU	4.0/4.0	0.6/0.8	3.8/4.0	0.5/0.0	3.5/3.3	0.6/0.5	3.1/3.0	0.4/0.6
TRUST	4.0/4.0	0.6/0.5	4.0/4.0	0.8/0.0	3.1/3.0	0.6/0.0	2.6/3.0	0.6/0.9

Abbreviations. ANX: anxiety; ATT: attitude toward technology; ENJ: perceived enjoyment; ITU: intention to use; PU: perceived usefulness; TRUST: trust.

Supplementary Table 21 Mean and median Almere model questionnaire (AMQ) construct scores at T0 and T6 in older adults, divided by living condition (ie, living alone or with somebody)

	Living alone				Living with somebody			
	T0<br>(N=8)		T6<br>(N=8)		T0<br>(N=12)		T6<br>(N=12)	
	Mean/median	SD/IQR	Mean/median	SD/IQR	Mean/median	SD/IQR	Mean/median	SD/IQR
ANX	3.1/3.0	1.1/1.9	3.8/4.0	0.6/0.6	4.3/5.0	1.2/0.8	3.9/4.0	1.1/1.0
ATT	3.9/3.8	0.6/0.5	3.6/3.7	0.5/0.3	4.4/4.3	0.4/0.8	3.7/3.7	0.7/1.6
ITU	3.2/3.3	0.8/1.2	3.2/3.0	0.9/0.6	4.3/5.0	1.0/1.2	3.8/3.8	1.1/2.1
ENJ	3.2/3.0	0.5/0.8	3.3/3.4	0.6/0.4	3.8/3.8	0.7/1.2	3.2/3.0	0.6/0.5
PU	3.3/3.3	0.5/0.1	3.3/3.2	0.6/0.8	4.1/4.0	0.6/0.5	3.6/4.0	0.6/1.0
TRUST	3.1/3.0	0.6/0.1	2.9/3.0	0.9/0.6	3.9/4.0	0.6/0.5	3.5/4.0	1.0/1.0

Abbreviations. ANX: anxiety; ATT: attitude toward technology; ENJ: perceived enjoyment; ITU: intention to use; PU: perceived usefulness; TRUST: trust.
